# Prohexadione calcium enhances rice growth and tillering under NaCl stress

**DOI:** 10.7717/peerj.14804

**Published:** 2023-02-06

**Authors:** Rongjun Zhang, Dianfeng Zheng, Naijie Feng, Quan-Sheng Qiu, Hang Zhou, Meiling Liu, Yao Li, Fengyan Meng, XiXin Huang, Anqi Huang, Yixiang Li

**Affiliations:** 1 Guangdong Ocean University, College of Coastal Agricultural Sciences, Zhanjiang, China; 2 South China, National Saline-tolerant Rice Technology Innovation Center, Zhanjiang, China; 3 Shenzhen Institute of Guangdong Ocean University, Shenzhen, China; 4 School of Life Sciences, Lanzhou University, MOE Key Laboratory of Cell Activities and Stress Adaptations, Lanzhou, Gansu, China

**Keywords:** Rice (*Oryza sativa L.*), NaCl stress, Pro-Ca, Tillering stage, Photosynthetic capacity

## Abstract

Salt stress affects crop quality and reduces crop yields, and growth regulators enhance salt tolerance of crop plants. In this report, we examined the effects of prohexadione-calcium (Pro-Ca) on improving rice (*Oryza sativa* L.) growth and tillering under salt stress. We found that NaCl stress inhibited the growth of two rice varieties and increased malondialdehyde (MDA) levels, electrolyte leakage, and the activities of the antioxidant enzymes. Foliar application of Pro-Ca reduced seedling height and increased stem base width and lodging resistance of rice. Further analyses showed that Pro-Ca application reduced MDA content, electrolyte leakage, and membrane damage in rice leaves under NaCl stress. Pro-Ca enhanced the net photosynthetic rate (*Pn*), stomatal conductance (Gs), and intercellular CO_2_ concentration (*Ci*) of rice seedlings, while increasing the activities of superoxide dismutase (SOD), catalase (CAT), peroxidase (POD), and ascorbic acid peroxidase (APX) at the tillering stage under salt stress. Overall, Pro-Ca improves salt tolerance of rice seedlings at the tillering stage by enhancing lodging resistance, reducing membrane damages, and enhancing photosynthesis and antioxidant capacities of rice seedlings.

## Introduction

As one of the main abiotic stresses, salt stress affects the growth and yield of crops (Gerona et al., 2019). According to incomplete statistics, more than 1/5 of the cultivated land in the world is threatened by salt stress (Zhang et al., 2012). At the same time, due to the rise of the groundwater level with high salinity and unreasonable irrigation and drainage, the global salinization area of cultivated land is increasing at a rate of 0.3−1.5 million hectares per year (Zhao et al., 2020a; Zhao et al., 2020b; Zhang et al., 2021). When the soil electrical conductivity (EC) reaches 4 dS/m, it is usually considered saline-alkali land (40 mM NaCl), which will produce an osmotic pressure of approximately 0.2 MPa, therefore causing side effects on the yield of most crops (Taratima, Chomarsa & Maneerattanarungroj, 2022). Salt stress reduces the absorption of water by plants by reducing osmotic potential, which makes plant cells swell and causes osmotic stress to plants (Ashraf & Foolad, 2007; Apse & Blumwald, 2002). Various transporters in plants play a role in the root system, causing ions to accumulate widely in the cytoplasm, affecting the absorption and distribution of nutrients by crops, destroying the integrity of the plasma membrane and the activity of antioxidant enzymes in cells, and finally affecting the yield of plants (Bhivare & Nimbalkar, 1984).

To cope with osmotic stress and ionic stress caused by salt stress on plant growth, plants have evolved various adaptive mechanisms to cope with salt stress. One of the main adaptive mechanisms is the accumulation of compatible solutes (Sharma & Dietz, 2006; Ashraf & Foolad, 2007). Plants maintain their cell homeostasis by increasing organic osmotic regulators such as proline and soluble sugar through osmotic regulation mechanisms, therefore protecting cells. Soluble sugar and proline, as osmotic regulators, protect plant cell homeostasis under salt stress by balancing the osmotic pressure of the cytosol and vacuole with that of the external environment (Elsheery et al., 2020). Additionally, salt stress will increase the content of reactive oxygen species (ROS) in plant cells (Hasegawa et al., 2000; Banu et al., 2009; Banu et al., 2010) and lead to an increase in lipid peroxidation in plant tissues, therefore inducing oxidative stress (Shabala & Pottosin, 2014; Naser et al., 2016). Plants have enzymatic and nonenzymatic antioxidant defense systems to protect cells from the destructive effects of ROS. The main antioxidant enzymes for scavenging ROS include CAT, guaiacol peroxidase (POX), and APX (Kibria et al., 2017). Nonenzymatic antioxidants include ascorbic acid, ascorbate (AsA), glutathione (GSH), phenolic compounds, and alpha-tocopherol. In addition, salt stress destroys plant metabolism and changes plant gene expression, which leads to the accumulation or consumption of some metabolites, resulting in an imbalance in cell protein levels (Lee et al., 2013). MDA is also often used as a suitable biomarker for lipid peroxidation under stress (Naser et al., 2016).

Rice is one of the main and most valuable food crops in the world (Tilman et al., 2011), the second largest cereal crop in the world after wheat (El Sayed et al., 2021), and the staple food of more than half of the world’s population and nearly 60% of China’s population (Zhao et al., 2020a; Zhao et al., 2020b; Wu et al., 2021). The yield of rice is mainly affected by the characteristics of aboveground structure, such as plant height, tiller number, tiller angle, leaf angle and panicle size (Wang & Li, 2008; Liu et al., 2020). Tillers grow independently of the mother stem through adventitious roots, which is an important determinant of panicle number (Wang & Jiao, 2018; Li et al., 2003), so the germination and growth of tillering buds are important agronomic traits that determine rice yield (Liu et al., 2020). Tiller angle, as a very important morphological feature of plant structure, affects ideal plant type and optimal planting density (Dong et al., 2016; Wang et al., 2021). Salinity will reduce the number of tillers in rice (Razzaque et al., 2009), and the primary and secondary tillers are more affected than the main stem, which usually leads to yield reduction (Ruan, Hu & Schmidhalter, 2008). Salt stress significantly reduced *Pn*, *Gs*, apparent mesophyll conductance (AMC), effective quantum yield of PSII photochemistry (Phi (PSII)), and electron transport rate (ETR) during tillering periods (Xu et al., 2019). The effect of salt stress on rice is closely related to the development stage, the severity and duration of stress and the variety, and rice can tolerate 3 dS/m salinity. At a salinity of 3.5 dS/m, rice yield decreased by 10%, while at a salinity of 7.2 dS/m, rice yield decreased by 50% (Taratima, Chomarsa & Maneerattanarungroj, 2022). Naim (2014) also proved that 50 mM, 100 mM, and 150 mM salt stress could reduce plant height, tiller, leaf relative water content (RWC), and water content of rice in the whole growth period.

Previous studies have been performed on the mechanism of plant stress tolerance and proved that exogenous substances can effectively reduce the damage caused by external stress to plant growth and development (Ashraf & Foolad, 2007; Hasanuzzaman et al., 2014). Parveen et al. (2021) proved that exogenous ABA can significantly reduce the damage to plants caused by salt stress. Exogenous foliar application of 6-BA before tillering bud germination can promote tillering bud germination by regulating endogenous hormones (Liu et al., 2011). The application of gibberellic acid 3 (GA3) increased the dry matter accumulation of tillers, improved the nitrogen metabolism of plants, and stimulated the tillering development of wheat at the tillering stage (Guoping, 1997). 4D and ethephon have also been shown to reduce the mortality of sugarcane tillers (Singh, Singh & Tiwari, 2018). Plant growth regulators, as new regulators, are an effective way to increase crop yield. It has been increasingly used in the regulation of plant tillering (Tanveer et al., 2018). As a new plant growth retardant, Pro-Ca can prevent the conversion of GA20 to GA1, maintain and prolong the activity level of existing gibberellin, reduce plant height and shorten internode length to resist lodging at a lower dose (Verma, Jain & Kaur, 2010). Nakayama et al. (1990) found that Pro-Ca can shorten the height of rice stalks and increase the chlorophyll content, therefore increasing the *Pn* of plants and promoting crop growth. Studies have proven that foliar application of Pro-Ca has significant effects on soybean, tomato and sweet potato (Feng et al., 2021; Soleimani Aghdam, 2013; Njiti et al., 2013). Pro-Ca can also increase the number of grains per spike, without residue and pollution, and has broad application prospects (Nakayama et al., 1990). However, the effects and mechanisms underlying the damage of rice tillering under salt stress remains to be studied.

In this study, the morphological changes of rice tillers and physiological characteristics of leaves under salt stress, as well as the protective effects of Pro-Ca on rice growth were studied.

## Material and Methods

### Materials and reagents

The seeds of inbred rice ‘Huanghuazhan’ (HHZ) were obtained from Longping Seed Co., Ltd. (Hunan, China). Seeds of the hybrid rice variety ‘Xiangliangyou900’ (X900) were collected from Nianfeng Seed Technology Co., Ltd. (Hunan, China).

The chemical reagent original solution (5% Pro-Ca) used in this experiment was provided by the College of Coastal Agricultural Sciences, Guangdong Ocean University. The regulator concentration was 100 mg L^−1^.

### Experimental designs

The soil culture experiment was conducted in July 2021 in the sunlight greenhouse of Guangdong Ocean University in a controlled environment (the day/night temperature is 30/28 °C, the day/night luminous period was 10/14 h, and the relative humidity was 60/70%). Rice seeds were sterilized with 2.5% sodium hypochlorite (NaClO) for 15 min. Subsequently, the seeds were soaked in distilled water at 30 °C for 24 h and transferred to the dark at 30 °C for germination for 24 h. The germinated seeds were spread evenly on the seedling tray (size 30 × 60 cm), with approximately 5–8 seeds per hole. The cultivated soil was treated before transplanting. Each plastic pot (diameter × bottom diameter × height of 19 × 15 × 18 cm) was filled with 3 kg of latosol. On the 7th day before transplanting rice seedlings (June 19th, 2021), 1 L of water was added to each pot to soak and stir the soil. Fertilization and stirring were carried out on the 2nd day before transplanting. After the water surface was stable, marking was made, and then water was regularly added to keep the water layer. When the seedlings reached the 3 leaf/1 heart stage, the seedlings were selected with consistent growth and were transplanted into plastic pots (June 26th, 2021). The transplanting depth was approximately 1.5 cm, with three holes per barrel. One plant was in one hole, and the spacing between the two holes was 10 cm. The leaf age was marked once every 5 days. After turning green (July 1st, 2021), 100 mg L^−1^ Pro-Ca was sprayed on the leaf surface before tillering. The spraying was made evenly on the front and back of each leaf to moisten it without dripping to ensure that it had absorbed completely. After 48 h of Pro-Ca treatment (July 3rd, 2021), 0.3% (approximately 2.92 g/L) NaCl was applied to the corresponding cultivated soil. The salt content of the water layer was monitored by a salinometer to ensure that the salt content of the water layer remains relatively stable throughout the whole growth process. There were four treatments for each variety: (1) Control (distilled water + 0% NaCl), (2) S (distilled water + 0.3% NaCl), (3) Pro-Ca (100 mg L^−1^ Pro-Ca + 0% NaCl), and (4) Pro-Ca+S (100 mg L^−1^ Pro-Ca + 0.3% NaCl). Each treatment was tested with four replicates. The samples were harvested after 7, 14, and 21 days (July 10th, 17th, and 24th, 2021) of NaCl treatment for the assays.

### Determination of morphological indices

The plants were harvested after 7, 14, and 21 days under salt stress for morphological index determination. The number of leaves with more than two branches was counted as a tiller, and the number of tillers was counted based on this standard. The fifth leaf sheath of the main stem outside the tiller was removed. The tiller bud morphology in the fifth leaf axil was measured. The plant height and root length were measured by the direct measurement method. A Vernier caliper was used to measure the stem base width at the junction of the stem and root. The leaf area of the functional leaf (the penultimate leaf and the third to last leaf) of each main stem was measured by a leaf area meter (YX-1241). The plant was rinsed with deionized water. After drying the water with filter paper, the fresh weights of the shoots and roots were measured. The samples were de-enzymed in an oven at 105 °C for 30 min and dried at 75 °C to constant weight to measure the dry weight of shoots and roots.

### Measurements of photosynthetic characteristics and SPAD of leaves

The *Pn*, transpiration rate (*Tr*), *Gs*, and intercellular CO_2_ concentration (*Ci*) of leaves were measured at 9:00–11:30 a.m. with a Li-6400 portable photosynthetic apparatus (LI-COR, Inc., USA) at 7, 14, and 21 days after NaCl stress. During the measurement, the conditions in the leaf chamber were as follows: photosynthetically active radiation (PAR) of 1,000 µmol m^−2^ s^−1^, leaf temperature of 34−38 °C, flow rate of 500 ml/s, and relative humidity of the reference chamber of 60–78%. The SPAD value of the penultimate leaf of the main stem was measured by a SPAD-502 portable chlorophyll meter (Konica Minolta, Japan) after 7, 14, and 21 days of salt stress.

### Determination of membrane damage index

Electrolyte leakage was measured according to the method described by Ahmad et al. (2016). Fresh leaves (0.1 g, penultimate leaf and third to last leaf) were soaked in 10 mL of deionized water at room temperature for 12 h. The conductivity (R1) was measured with a conductivity meter. The samples were boiled in boiling water for 30 min, and the conductivity (R2) was measured after cooling. The blade’s relative conductivity was calculated according to the formula: electrolyte leakage = R1/R2 × 100% (Chen et al., 2022). The malondialdehyde (MDA) content was determined by the TBA method. The leaves (0.5 g) were ground in liquid nitrogen, added to 10 ml phosphate buffer (10% TCA), ground into homogenate, and centrifuged at 6,000 × g for 20 min. One milliliter of the supernatant was mixed with 2 ml of 0.6% TBA (thiobarbituric acid) in a centrifuge tube. The mixture was boiled in a boiling water bath for 15 min and centrifuged at 4,000 × g for 20 min. The absorbance of the supernatant was measured at 450 nm, 532 nm, and 600 nm. The MDA content was estimated on the method provided by Guo et al. (2018).

### Determination of antioxidative enzyme activities

At 7, 14, and 21 days after NaCl stress, the functional leaves of the main stem of rice were quickly frozen in liquid nitrogen and then stored in the refrigerator at −80 °C. The leaves (0.5 g) were ground in liquid nitrogen, and then 10 ml precooled phosphate buffer (0.05 mM PBS, pH 7.8) was added, ground into homogenate, and centrifuged at 4 °C and 6,000 × g for 20 min. The supernatant was taken to measure the activities of superoxide dismutase (SOD), catalase (CAT), peroxidase (POD), and ascorbate peroxidase (APX). SOD activities were determined by using nitro blue tetrazolium (NBT). Then, 0.1 ml of enzyme solution was added to 2.9 ml of the reaction mixture (2.61 ml met + 0.097 ml EDTA-Na_2_ + 0.097 ml NBT + 0.097 ml riboflavin), and the mixture was irradiated with 4,000 × g at 25 °C for 20 min. The absorbance at 560 nm was measured, and the total activities of SOD were calculated by the method of Giannopolitis & Ries (1977). Then, 0.1 ml of enzyme solution was mixed with 2.9 reaction solution (PBS pH 7.0 + 30% H_2_O_2_) to measure the dynamic absorbance at 240 nm, which was recorded 4 times every 30 s. The activities of CAT were calculated on the method provided by Aebi (1984). Three milliliters of reaction solution (PBS pH 6.0+ guaiacol) was mixed with 40 microliters of enzyme solution. The dynamic absorbance at 470 nm was measured and recorded 4 times every 30 s. POD activity was calculated according to the method reported by Klapheck, Zimmer & Cosse (1990). Then, 0.1 ml of enzyme solution was mixed with the reaction solution (2.6 ml EDTA-Na_2_ + 0.15 ml ASA + 0.15 ml H_2_O_2_), and the dynamic absorbance was measured at 290 nm every 30 s at 20 °C 4 times to calculate the APX activity by the procedure described in Nakano & Asada (1981).

### Statistical analyses

The data are presented as the mean ± standard error of the mean (SEM). Statistical significance was determined by one-way analysis of variance (ANOVA) with Duncan’s comparison tests at *P* < 0.05. Figures and charts were prepared using Origin 2021. Correlation analysis was performed with SPSS (25.0, IBM Corp., Armonk, NY, USA) and Origin 2021.

## Results

### Effects of Pro-Ca on morphological parameters of rice at the tillering stage under NaCl stress

**Figure 1:**
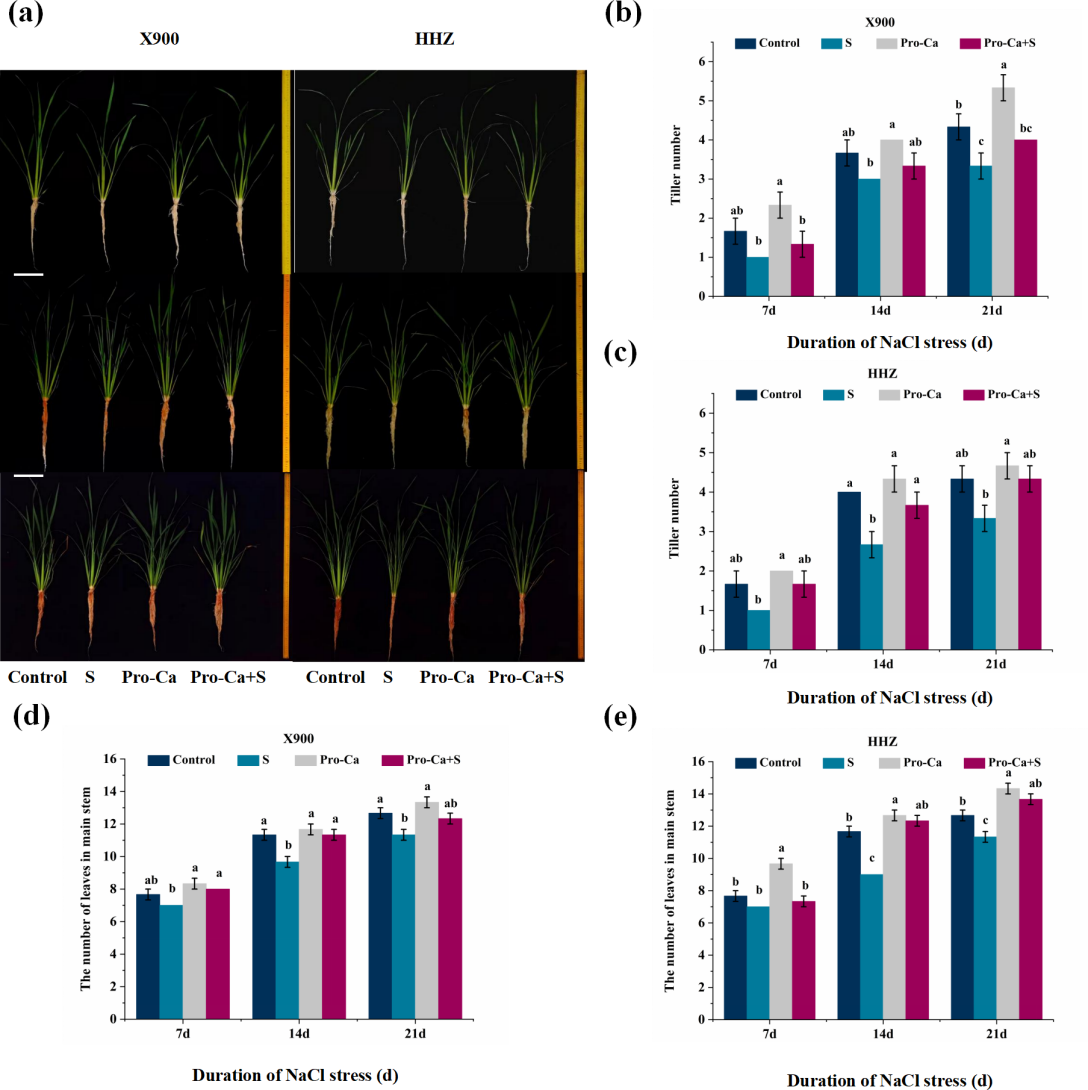
Effects of Pro-Ca on rice growth under NaCl stress (left: X900; right: HHZ). (A) Plant growth. Rice morphology after 7, 14, and 21d of salt stress. (B–E) Tiller numbers and leaf numbers in the main stem. Comparison of tiller numbers and leaf numbers in the main stem between different treatments. Values are means ± SD (*n* = 3) and bars indicate SD. Columns with different letters indicate significant difference at *P* < 0.05 (Duncan’s test). CK (distilled water + 0% NaCl), S (distilled water + 0.3% NaCl), Pro-Ca (100 mg L^−1^ Pro-Ca + 0% NaCl) and Pro-Ca +S (100 mg L^−1^ Pro-Ca + 0.3% NaCl).

**Figure 2:**
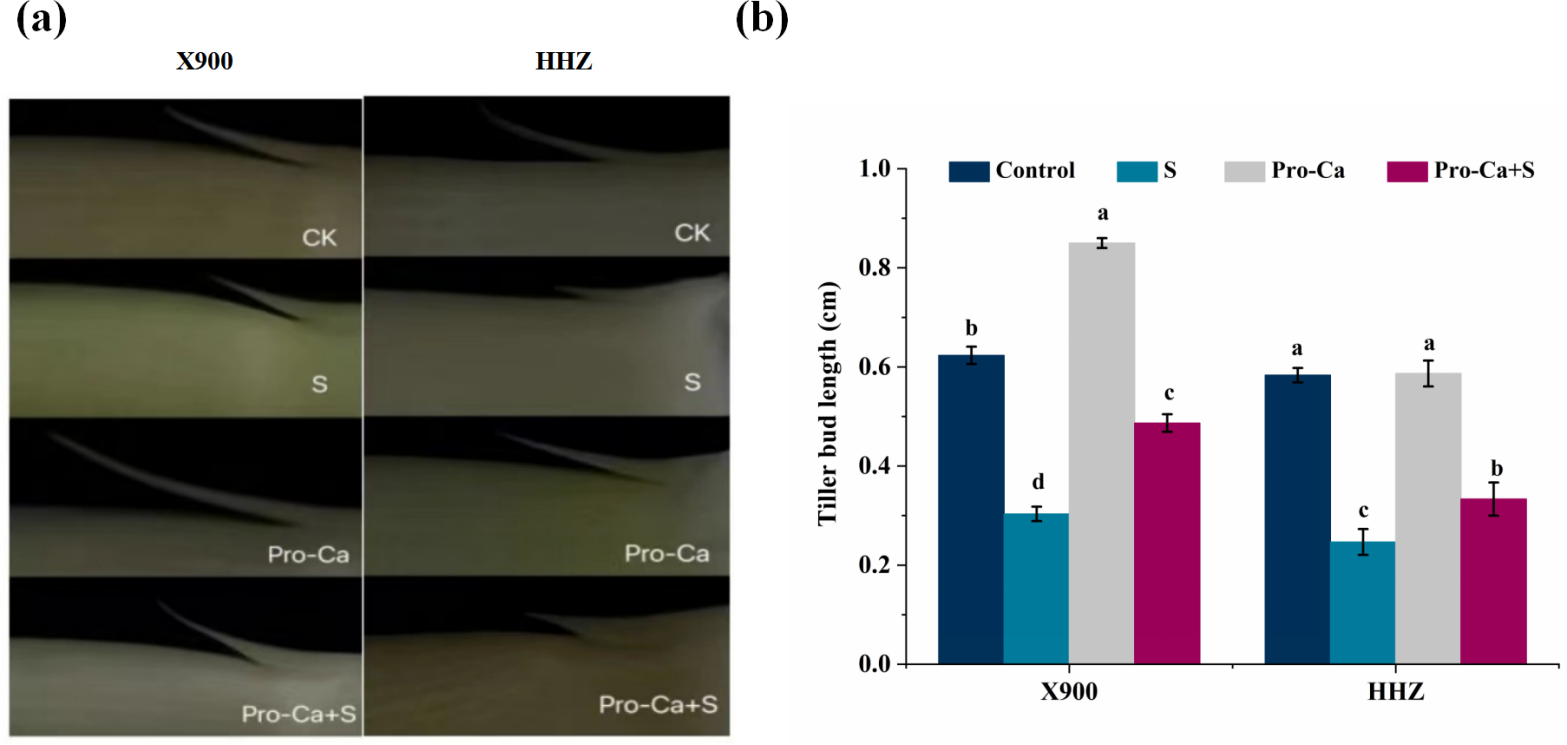
Effect of Pro-Ca on tillering buds under NaCl stress. (A) Tillering buds. Tillering buds in the fifth axil of rice under NaCl stress. (B) The length of tillering buds. The length of tillering buds in the fifth leaf axil. The different letters are significant differences according to Duncan’s new multiple range test (*P* < 0.05) based on one-way ANOVA.

From the experimental results, we can see that NaCl stress has a negative impact on the growth of the two rice varieties. NaCl stress reduced the tiller number of X900 by 40.00%, 18.18%, and 23.08% on the 7th, 14th, and 21st days, respectively, while that of HHZ decreased by 40.00%, 33.33%, and 23.08% under NaCl stress, respectively (Figs. 1B and 1C). Compared with the control, NaCl stress significantly reduced the tillering bud length, tillering angle, tillering number, the number of leaves in the main stems and the fifth leaf axils of the two rice varieties after NaCl stress. As shown in Fig. 2, the tillering bud length of X900 and HHZ was inhibited by 51.34% and 57.71% on the 7th day after NaCl stress, respectively. Similarly, Table 1 shows that NaCl stress reduced the tillering angle of X900 by 62.50%, 50.02%, and 17.65% on the 7th, 14th, and 21st days, respectively. NaCl stress reduced the tillering angle of HHZ by 46.68%, 22.23%, and 14.27% on the 7th, 14th, and 21st days, respectively. Additionally, we found that the tillering angles of the two varieties decreased but not significantly after 21 days of NaCl stress. NaCl stress also significantly reduced the number of leaves in the main stem of the two rice varieties on the 14th and 21st days. The number of leaves and main stem of X900 decreased by 14.71% and 10.53% on the 14th and 21st days, and HHZ decreased by 22.86% and 10.53%, respectively (Figs. 1D and 1E).

**Table 1:** Effects of Pro-Ca on tiller angle, plant height and leaf area at the tillering stage under NaCl stress.

Index	Treatments	X900		
		7	14	21
Tiller angle	Control	26.7 ± 1.7a	26.7 ± 3.3a	28.3 ± 1.7a
	S	10.0 ± 0.0b	13.3 ± 3.3b	23.3 ± 3.3a
	Pro-Ca	23.3 ± 3.3a	26.7 ± 3.3a	30.0 ± 0.0a
	Pro-Ca+S	23.3 ± 3.3a	23.3 ± 3.3ab	26.7 ± 3.3a
Plant height	Control	55.8 ± 0.3a	62.8 ± 0.3a	73.2 ± 0.1a
	S	48.6 ± 0.2b	57.4 ± 0.2b	66.8 ± 0.5b
	Pro-Ca	44.2 ± 0.1d	49.8 ± 0.3d	63.4 ± 0.2d
	Pro-Ca+S	47.5 ± 0.1c	52.6 ± 0.1c	65.6 ± 0.1c
Leaf area	Control	3963.4 ± 157.4ab	6009.9 ± 365.9a	6891.7 ± 120.9a
	S	3415.5 ± 14.5b	3991.0 ± 143.4b	4988.1 ± 114.1d
	Pro-Ca	4365.3 ± 401.0a	5900.1 ± 305.8a	6227.3 ± 126.0b
	Pro-Ca+S	3904.5 ± 7.3ab	5323.7 ± 48.2a	5808.2 ± 5.6c

		HHZ		
		7	14	21
Tiller angle	Control	25.0 ± 2.9ab	30.0 ± 0.0a	23.3 ± 3.3a
	S	13.3 ± 3.3b	23.3 ± 1.7b	20.0 ± 0.0a
	Pro-Ca	28.3 ± 4.4a	30.0 ± 0.0a	26.7 ± 3.3a
	Pro-Ca+S	23.3 ± 3.3ab	28.3 ± 1.7a	23.3 ± 3.3a
Plant height	Control	54.0 ± 0.1a	61.6 ± 0.2a	74.8 ± 0.2a
	S	48.3 ± 0.2b	56.1 ± 0.3b	66.2 ± 0.1b
	Pro-Ca	42.9 ± 0.4d	50.0 ± 0.1d	64.0 ± 0.3d
	Pro-Ca+S	45.2 ± 0.3c	53.9 ± 0.2c	65.4 ± 0.2c
Leaf area	Control	3569.1 ± 86.6a	4956.6 ± 284.3ab	5844.9 ± 90.6a
	S	2273.1 ± 131.4b	3318.9 ± 201.3c	3440.1 ± 156.9c
	Pro-Ca	3849.6 ± 82.3a	5625.2 ± 227.4a	6314.1 ± 222.7a
	Pro-Ca+S	3661.7 ± 28.0a	4620.6 ± 132.9b	5013.8 ± 57.1b

**Notes.**

Values described are the means ± SE (*n* = 3). Different letters denote significant difference from Duncan’s LSD test (*p* < 0.05).

**Figure 3:**
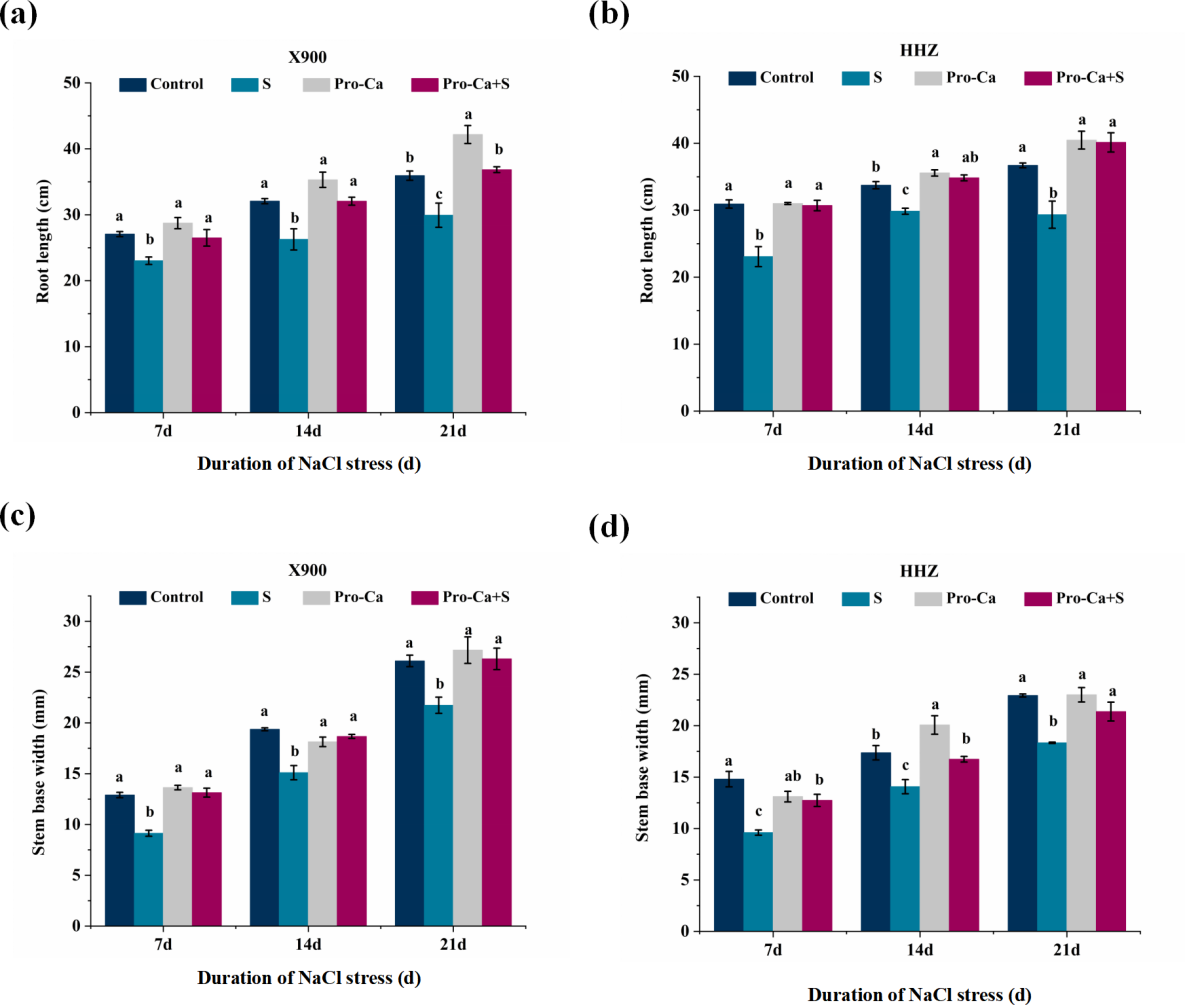
Effects of Pro-Ca on rice morphological index under NaCl stress. (A and B) Root length. Root length of X900 (A) and HHZ (B) after 7, 14, and 21 d under NaCl stress. (C and D) Stem base width. Stem base width of X900 (C) and HHZ (D) after 7, 14, and 21d under NaCl stress. The different letters are significant differences according to Duncan’s new multiple range test (*P* < 0.05) based on one-way ANOVA.

In comparison with the control, NaCl stress also significantly inhibited the plant height, root length, stem base width, and leaf area of the two rice varieties. Among them, NaCl stress significantly inhibited the plant height of X900 and HHZ, which of X900 decreased by 12.96%, 8.60%, and 8.70% on the 7th, 14th, and 21st days after NaCl stress, and the plant height of HHZ decreased by 10.56%, 10.56%, and 11.41%, respectively (Table 1). As shown in Figs. 3A and 3B, the root length of X900 was inhibited by 14.90%, 18.09%, and 16.70%, and the root length of HHZ was shortened by 25.43%, 11.52%, and 20.07% under NaCl stress on the 7th, 14th, and 21st days, respectively. Salt stress also inhibited the stem base width of X900 by 29.20%, 22.03%, and 16.73% on the 7th, 14th, and 21st days, respectively. On the 7th, 14th, and 21st days, the stem base width of HHZ was inhibited by 35.14%, 19%, and 20.06%, respectively (Figs. 3C and 3D). In comparison with S, the leaf area of X900 rice decreased by 13.82%, 33.59%, and 27.62% on the 7th, 14th, and 21st days after salt stress, and HHZ decreased by 36.31%, 33.04%, and 41.14%, respectively (Table 1).

As shown in Table 2, NaCl stress also significantly inhibited the fresh weight and dry weight of rice above- and belowground. Under NaCl stress, the aboveground dry weight of X900 decreased by 35.13%, 17.13%, and 12.60% on the 7th, 14th, and 21st days, respectively, and that of HHZ decreased by 39.58%, 35.30%, and 27.75%, respectively, when compared with the control. The underground dry weight of X900 decreased by 58.15%, 42.82%, and 28.42%, and that of HHZ decreased by 55.10%, 31.98%, and 52.99% on the 7th, 14th, and 21st days after NaCl stress, respectively.

**Table 2:** Effects of Pro-Ca on shoot fresh weight, root fresh weight, shoot dry weight and root dry weight at the tillering stage under NaCl stress.

Index	Treatments	X900		
		7	14	21
Shoot fresh weight	Control	3.4786 ± 0.4465b	8.649 ± 0.1177b	15.6598 ± 0.1894a
	S	2.7319 ± 0.0559b	7.1282 ± 0.0501c	12.2794 ± 0.3021c
	Pro-Ca	4.4262 ± 0.2424a	10.3236 ± 0.5460a	16.1107 ± 0.1469a
	Pro-Ca+S	3.5562 ± 0.1664ab	8.1891 ± 0.1335b	14.8181 ± 0.1266b
Root fresh weight	Control	1.3994 ± 0.0224b	4.4791 ± 0.2167b	7.5917 ± 0.1484b
	S	1.0274 ± 0.0240c	2.4569 ± 0.0417d	5.4239 ± 0.0269d
	Pro-Ca	1.5774 ± 0.0522a	5.2803 ± 0.0622a	8.8845 ± 0.1750a
	Pro-Ca+S	1.342 ± 0.0049b	4.1016 ± 0.0195c	6.821 ± 0.1524c
Shoot dry weight	Control	0.579 ± 0.0078b	1.2507 ± 0.0093b	2.0538 ± 0.0198b
	S	0.3756 ± 0.0097d	1.0365 ± 0.0320d	1.7951 ± 0.0225d
	Pro-Ca	0.7106 ± 0.0074a	1.4324 ± 0.0230a	2.4521 ± 0.0207a
	Pro-Ca+S	0.5298 ± 0.0016c	1.1722 ± 0.0217c	1.8911 ± 0.0210c
Root dry weight	Control	0.1355 ± 0.0007b	0.4757 ± 0.0076b	0.9000 ± 0.0098b
	S	0.0567 ± 0.0058d	0.272 ± 0.0111c	0.6442 ± 0.0055c
	Pro-Ca	0.1471 ± 0.0025a	0.619 ± 0.0135a	1.1151 ± 0.0625a
	Pro-Ca+S	0.1104 ± 0.0006c	0.4451 ± 0.0044b	0.8611 ± 0.0052b

		HHZ		
		7	14	21
Shoot fresh weight	Control	3.6741 ± 0.0197b	7.8301 ± 0.0837b	15.6625 ± 0.1934a
	S	2.0782 ± 0.0653d	5.3581 ± 0.2280d	10.5445 ± 0.1707c
	Pro-Ca	3.9324 ± 0.1285a	9.1533 ± 0.1251a	16.2861 ± 0.2961a
	Pro-Ca+S	2.9625 ± 0.0066c	7.1635 ± 0.2685c	13.792 ± 0.0294b
Root fresh weight	Control	1.5611 ± 0.0101b	3.7778 ± 0.1082b	7.8132 ± 0.1301b
	S	0.7775 ± 0.0188d	2.1276 ± 0.0478d	4.1575 ± 0.0685d
	Pro-Ca	1.6481 ± 0.0133a	4.2903 ± 0.0847a	8.4824 ± 0.0314a
	Pro-Ca+S	1.4977 ± 0.0082c	3.3479 ± 0.0579c	6.3662 ± 0.0605c
Shoot dry weight	Control	0.569 ± 0.0299b	1.2344 ± 0.0069b	2.4295 ± 0.0457b
	S	0.3438 ± 0.0130d	0.7987 ± 0.0096d	1.7554 ± 0.0318d
	Pro-Ca	0.6374 ± 0.0097a	1.4048 ± 0.0274a	2.7007 ± 0.0344a
	Pro-Ca+S	0.4865 ± 0.0092c	1.1452 ± 0.0169c	2.2801 ± 0.0453c
Root dry weight	Control	0.157 ± 0.0007b	0.3655 ± 0.0022b	0.967 ± 0.0255b
	S	0.0705 ± 0.0024d	0.2486 ± 0.0099d	0.4546 ± 0.0118d
	Pro-Ca	0.1761 ± 0.0018a	0.4012 ± 0.0016a	1.0468 ± 0.0189a
	Pro-Ca+S	0.1441 ± 0.0015c	0.3211 ± 0.0011c	0.8328 ± 0.0197c

**Notes.**

Values described are the means ± SE (*n* = 3). Different letters denote significant difference from Duncan’s LSD test (*p* < 0.05).

Exogenous foliar application of Pro-Ca effectively alleviated the inhibition of NaCl stress on the growth parameters of the two varieties. Compared with S treatment, foliar application of Pro-Ca restored the length of the fifth axillary tiller bud of X900 and HHZ by 57.31% and 25.73%, respectively, after 7 days of NaCl stress (Fig. 2). The tillering angle of X900 was restored by 79.96%, 74.96%, and 66.80% on the 7th, 14th, and 21st days, respectively, and that of HHZ was restored by 85.69%, 74.96%, and 100% on the 7th, 14th, and 21st days, respectively, in the Pro-Ca+S treatment relative to S (Table 1). Foliar application of Pro-Ca restored the inhibition of the tiller number of X900 by 49.99%, 49.99%, and 66.67% on the 7th, 14th, and 21st days, which of HHZ was relieved by 100%, 75%, and 100%, respectively, when compared with S (Figs. 1B and 1C). Exogenous foliar application of Pro-Ca also alleviated the inhibition of salt stress on the number of main stem leaves, among which the number of main stem leaves of X900 was relieved by 149.99%, 100%, and 75.00% on the 7th, 14th, and 21st days, respectively, and HHZ was relieved by 49.99%, 125%, and 175%, respectively (Figs. 1D and 1E).

Exogenous foliar application of Pro-Ca under NaCl stress also significantly alleviated the inhibition of salt stress on root length, stem base width and leaf area of X900 and HHZ. The root length of X900 was relieved by 85.95%, 99.88%, and 115% on the 7th, 14th, and 21st days, respectively, and the root length of HHZ was restored by 97.03%, 128.30%, and 146.61%, respectively, in the Pro-Ca+S treatment relative to S (Figs. 3A and 3B). Compared with S treatment, foliar application of Pro-Ca under NaCl stress restored the stem base width of X900 and HHZ by 106.19%, 83.59%, 104.58%, 60.26%, 80.81%, and 65.94% on the 7th, 14th, and 21st days, respectively, with significant differences (Figs. 3C and 3D). Under NaCl stress, foliar application of Pro-Ca significantly relieved the leaf area of the two varieties, and the leaf area of X900 was relieved by 89.26%, 66.01%, and 43.08%, respectively, while that of HHZ was relieved by 107.14%, 79.48%, and 65.44%, respectively (Table 1).

It is worth noting that Pro-Ca, as a chlormequat chloride, can significantly reduce the plant height of rice when sprayed alone. From Table 1, we can see that compared with the control, the plant height of X900 sprayed with Pro-Ca decreased by 20.84%, 20.65%, and 13.35% on the 7th, 14th, and 21st days after NaCl stress, respectively. The plant height of HHZ decreased by 20.49%, 18.79%, and 14.44%, respectively. In addition, the aboveground biomass and underground biomass of the two rice varieties were significantly relieved under NaCl stress (Table 2).

### Effects of Pro-Ca on photosynthetic capacity at the rice tillering stage under NaCl stress

**Figure 4:**
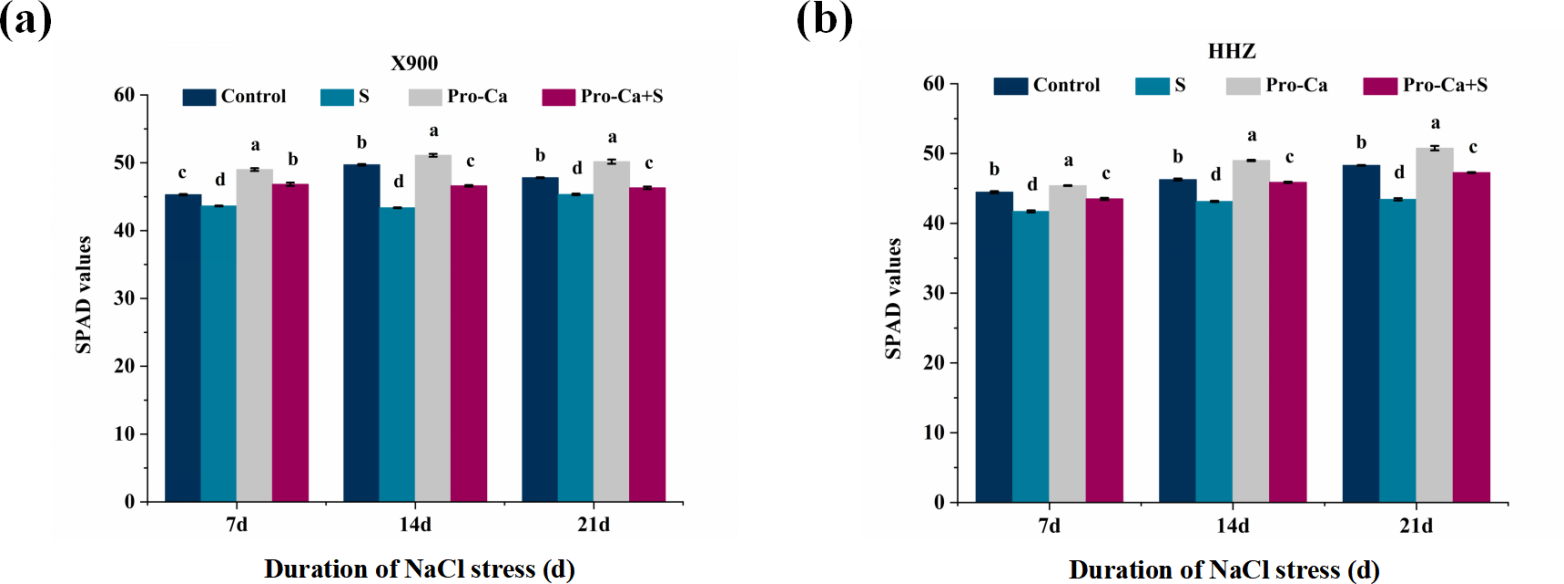
Effects of Pro-Ca on SPAD of rice under NaCl stress. (A and B) SPAD. SPAD of X900 (A) and HHZ (B) was measured with a portable chlorophyll meter SPAD-502 (Konica Minolta, Tokyo, Japan) after 7, 14, and 21 days of salt stress. The different letters are significant differences according to Duncan’s new multiple range test (*P* < 0.05) based on one-way ANOVA.

As shown in Fig. 4, under NaCl stress, the SPAD value of X900 decreased by 3.68%, 12.74%, and 5.16% on the 7th, 14th, and 21st days, respectively, and that of HHZ decreased by 6.22%, 6.77%, and 10.08%, respectively. Compared with S, the SPAD value of X900 was reduced by 192%, 51.05%, and 39.19% by foliar application of Pro-Ca externally, and the SPAD values of HHZ were relieved by 65.06%, 87.23%, and 78.77%.

**Figure 5:**
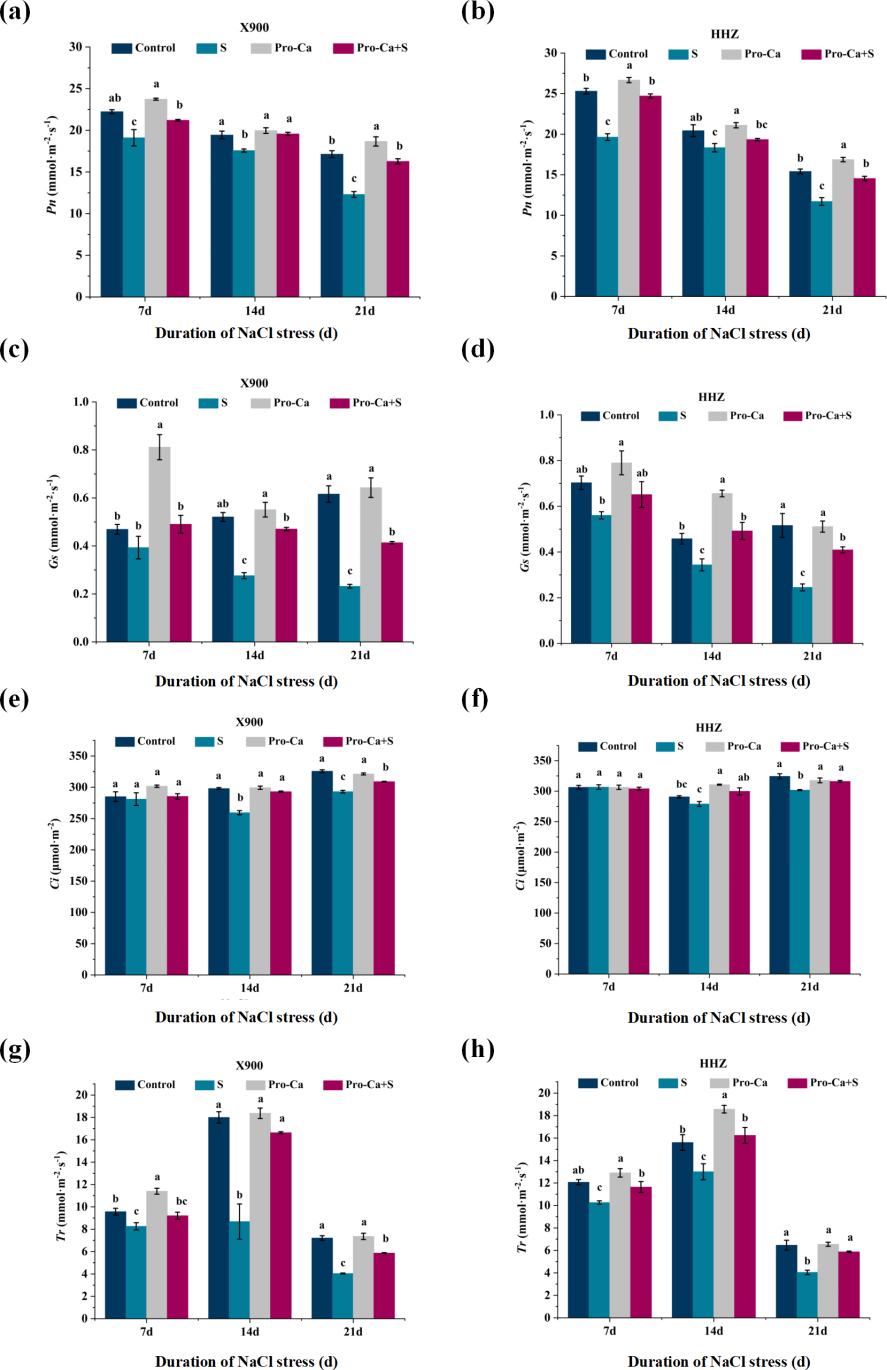
Effects of Pro-Ca on *Pn*, *Gs*, *Ci*, and *Tr* of rice under NaCl stress. (A and B) Net photosynthetic rate. *Pn* of X900 (A) and HHZ (B) was measured at 9:00–11:30 a.m. with Li-6400 portable photosynthetic apparatus (LI-COR, Inc., USA) on 7, 14, and 21 days after NaCl stress. (C and D) Stomatal conductance. *Gs* of X900 (c) and HHZ (d) were measured at 9:00–11:30 a.m. with Li-6400 portable photosynthetic apparatus (LI-COR, Inc., USA) on 7, 14, and 21 days after NaCl stress. (E and F) Intercellular CO_2_ concentration. *Ci* of X900 (e) and HHZ (f) was measured at 9:00–11:30 a.m. with Li-6400 portable photosynthetic apparatus (LI-COR, Inc., USA) on 7, 14, and 21 days after NaCl stress. (G and H) Transpiration rate. *Tr* of X900 (G) and HHZ (H) was measured at 9:00–11:30 a.m. with Li-6400 portable photosynthetic apparatus (LI-COR, Inc., USA) on 7, 14, and 21 days after NaCl stress. The different letters are significant differences according to Duncan’s new multiple range test (*P* < 0.05) based on one-way ANOVA.

Under NaCl stress, the photosynthetic capacity of both varieties decreased significantly. Compared with the control, the *Pn* of X900 decreased by 14.09%, 9.61%, and 28.21% on the 7th, 14th, and 21st days after NaCl stress, respectively, while that of HHZ decreased by 22.40%, 10.28%, and 24.03%, respectively (Figs. 5A and 5B). As shown in Figs. 5C and 5D, compared with the control, the *Gs* of X900 decreased by 46.99% and 62.39% on the 14th and 21st days and that of HHZ decreased by 24.96% and 52.52% on the 14th and 21st days, respectively. The *Gs* of the two varieties decreased by 16.20% and 20.30% on the 7th day, but not significantly. Under NaCl stress, the *Ci* of X900 decreased significantly by 12.98% and 10.03% on the 14th and 21st days, respectively. Compared with the control, the *Ci* of HHZ decreased by 4.01% and 6.99% on the 14th and 21st days after NaCl stress, respectively, and the *Tr* of the two rice varieties also decreased significantly on the 7th, 14th and 21st days after NaCl stress (Figs. 5E–5H).

As shown in Fig. 5, the results of the Pro-Ca+S treatment showed that the *Pn* of X900 was relieved by 67.02%, 107.15%, and 82.07% and that of HHZ was relieved by 89.41%, 47.62%, and 76.58% at the 7th, 14th, and 21st days after exogenous foliar application of Pro-Ca under NaCl stress at the tillering stage. Exogenous foliar application of Pro-Ca also significantly alleviated the inhibition of NaCl stress on *Gs*, among which the *Gs* of X900 was relieved by 79.40%, and 47.25% on the 14th and 21st day, and that of HHZ was relieved by 129.75% and 60.63%, respectively, on the 7th day, *Gs* of the two varieties was relieved but not significantly (Figs. 5C and 5D). On the 7th, 14th, and 21st days, the *Ci* of X900 sprayed with exogenous Pro-Ca was relieved by 108.33%, 87.07%, and 48.98%, and the *Ci* of HHZ was relieved by 799.85%, 177.14%, and 63.24%, respectively, when compared with S (Figs. 5E and 5F). Exogenous foliar application of Pro-Ca alleviated the inhibition of salt stress on *Tr*, among which X900 alleviated *Tr* by 72.52%, 85.33%, and 57.56%, and HHZ alleviated *Tr* by 76.06%, 124.36%, and 75.62% on the 7th, 14th, and 21st days, respectively (Fig. 5G and 5H).

### Effects of Pro-Ca on the indices of membrane damage at the rice tillering stage under NaCl stress

**Figure 6:**
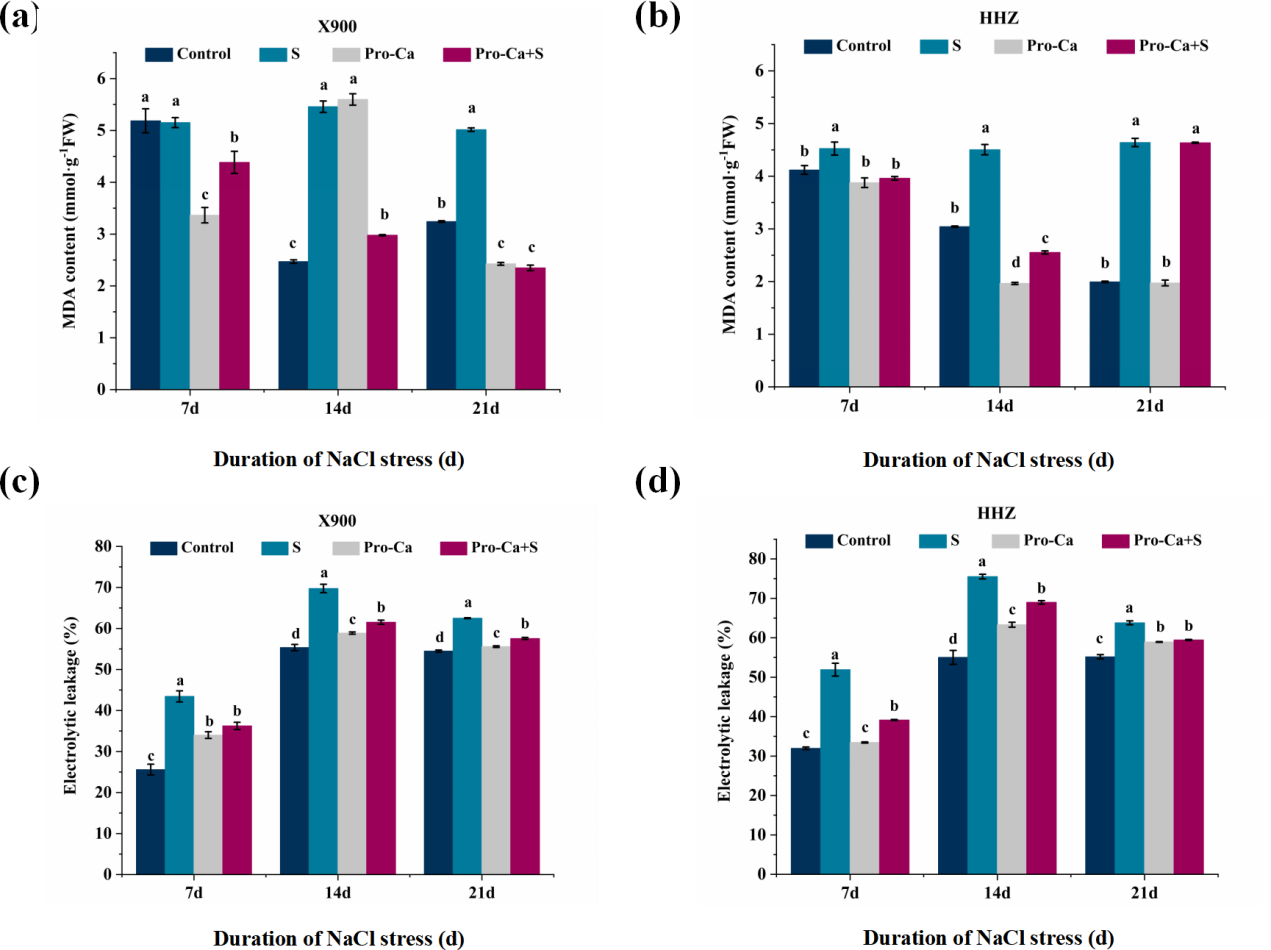
Effects of Pro-Ca on the indexes of membrane damage of rice under NaCl stress. (A and B) MDA contents. Contents of MDA of X900 (A) and HHZ (B) on 7, 14, and 21 days after NaCl stress. (C and D) Electrolyte leakage. Electrolyte leakages of X900 (C) and HHZ (D) were measured after 7, 14, and 21 days of salt stress. The different letters are significant differences according to Duncan’s new multiple range test (*P* < 0.05) based on one-way ANOVA.

The MDA content and electrolyte leakage of rice leaves at the tillering stage showed changes under NaCl stress. Compared with the control, the MDA content of X900 leaves increased by 121.05% and 54.73% on the 14th and 21st days, and the MDA content of HHZ decreased by 9.87%, 48.04%, and 132.81%, respectively, under NaCl stress (Figs. 6A and 6B). Similarly, compared with the control, the electrolyte leakage rate of X900 increased by 69.73%, 26.05%, and 14.74% on the 7th, 14th, and 21st days, respectively, and the electrolyte leakage rate of HHZ increased by 62.42% and 37.22%, respectively, under NaCl stress.

Compared with S treatment, exogenous foliar application of Pro-Ca alleviated the effect of NaCl stress on MDA, in which the MDA of X900 was relieved by 82.96% and 150.33% on the 14th and 21st days, respectively, and HHZ was relieved by 138.93% and 133.53% on the 7th and 14th days, respectively, which decreased on the 21st day, but the difference was not significant. On the 7th, 14th, and 21st days, the electrolyte leakage rate of X900 was relieved by 40.31%, 57.11%, and 61.80%, respectively, and HHZ was relieved by 64.04%, 31.87%, and 50.50%, respectively, in Pro-Ca+S when compared to S.

### Effects of Pro-Ca on antioxidant enzymes at the rice tillering stage under NaCl stress

**Figure 7:**
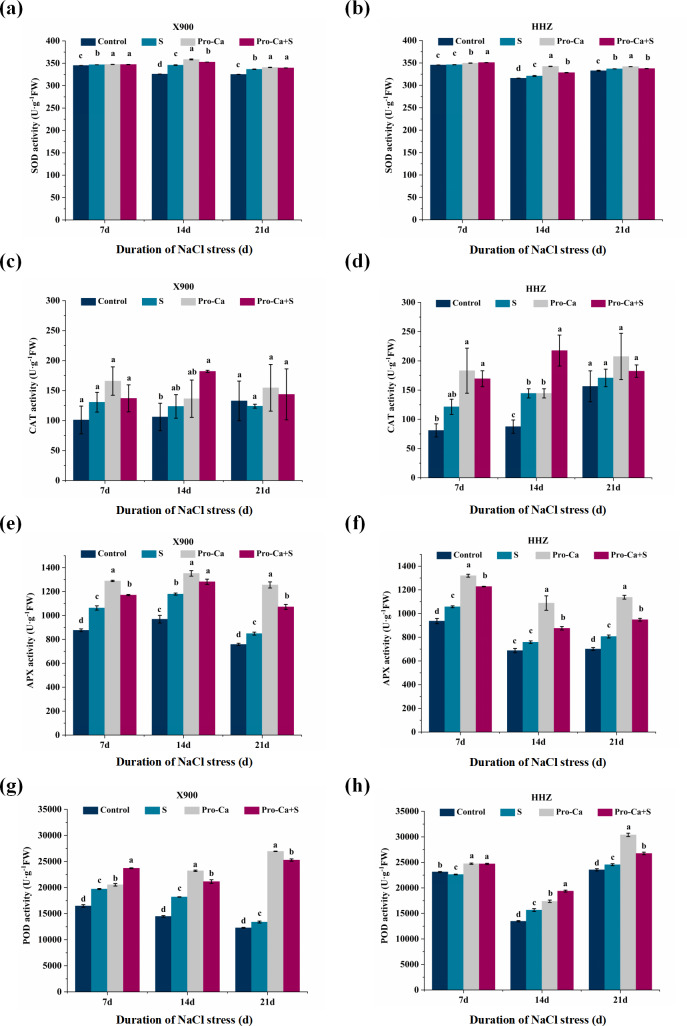
Effects of Pro-Ca on the indexes of the antioxidative enzyme activities of rice under NaCl stress. (A and B) The activities of SOD. Effects of Pro-Ca on the activities of SOD of X900 (A) and HHZ (B) on 7, 14, and 21 days after NaCl stress. (C and D) The activities of CAT. Effects of Pro-Ca on the activities of CAT of X900 (C) and HHZ (D) after 7, 14, and 21 days of salt stress. (E and F) The activities of APX. Effects of Pro-Ca on the activities of APX of X900 (E) and HHZ (F) on 7, 14, and 21 days after NaCl stress. (G and H) The activities of POD. Effects of Pro-Ca on the activities of POD of X900 (G) and HHZ (H) after 7, 14, and 21 days of salt stress. The different letters are significant differences according to Duncan’s new multiple range test (*P* < 0.05) based on one-way ANOVA.

As shown in Figs. 7A and 7B, compared with the control, salt stress increased the SOD activities of X900 by 0.50%, 6.13%, and 3.53% on the 7th, 14th, and 21st days, and the activity of SOD of HHZ increased by 0.18%, 1.55%, and 1.26%, respectively. Compared with the S treatment, in the Pro-Ca+S treatment, the SOD activities of X900 increased by 0.15%, 1.96%, and 0.88% on the 7th, 14th, and 21st days, respectively, and those of HHZ increased by 1.39%, 2.33%, and 0.20%, respectively. Under NaCl stress, the CAT activities of X900 and HHZ increased, and X900 increased by 29.39% and 16.60% on the 7th and 14th days, respectively, but it was not significant on the 7th day. HHZ increased by 49.88% and 65.03% on the 7th and 14th days and 9.21% on the 21st day, respectively. Similarly, exogenous foliar application of Pro-Ca under NaCl stress effectively increased CAT activity at the tillering stage. Compared with S, on the 7th, 14th and 21st days, the CAT activity of X900 leaves increased by 4.90%, 47.25% and 15.81%, respectively, and that of HHZ increased by 39.59%, 50.69% and 6.79%, respectively (Figs. 7C and 7D). NaCl stress (0.3%) also increased the activities of APX and POD in the two rice varieties. As shown in Figs. 7E and 7F, compared with the control, under NaCl stress, the APX activities of X900 increased by 21.32%, 21.68%, and 11.95% on the 7th, 14th, and 21st days, respectively, and those of HHZ increased by 12.90%, 10.29%, and 15.08%, respectively. Similarly, compared with the control, under NaCl stress, the POD activities of X900 increased by 19.67%, 25.79%, and 9.00% on the 7th, 14th, and 21st days, respectively, while HHZ increased by 16.26% and 4.24% on the 14th and 21st days, respectively (Figs. 7G and 7H). Exogenous foliar application of Pro-Ca on the surface of X900 and HHZ at the tillering stage under NaCl stress effectively increased the APX and POD activities of X900 and HHZ; on the 7th, 14th, and 21st days, the APX of X900 increased by 10.15%, 8.75%, and 26.34%, and the APX activities of HHZ leaves increased by 16.17%, 15.46%, and 17.59%, respectively (Figs. 7E and 7F). Exogenous foliar application of Pro-Ca also increased the POD activity of rice leaves under salt stress, in which the POD activities of X900 increased by 20.23%, 16.21%, and 88.87% and HHZ increased by 9.20%, 23.74%, and 8.98% on the 7th, 14th, and 21st days, respectively (Figs. 7G and 7H).

### Effects of Pro-Ca on osmotic substances at the rice tillering stage under NaCl stress

**Figure 8:**
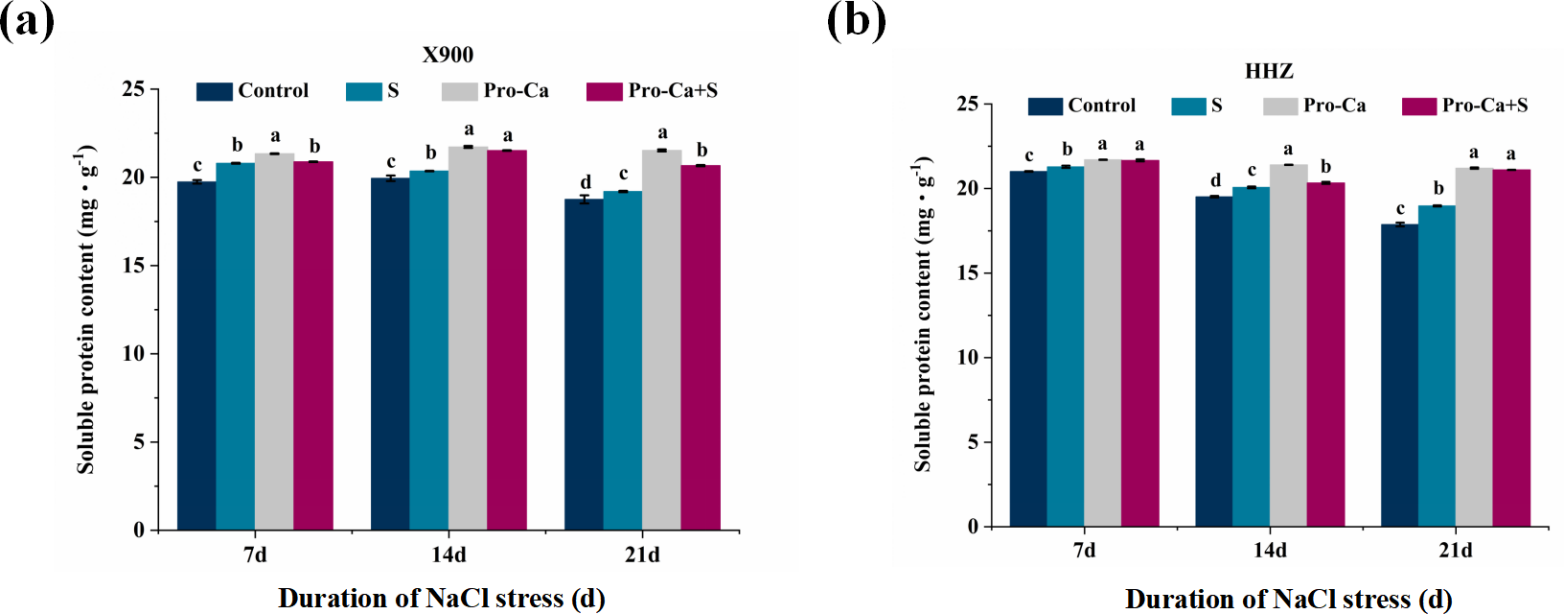
Effects of Pro-Ca on the osmotic substance of rice under NaCl stress. (A and B) Soluble proteins. Soluble protein contents in leaves of X900 (A) and HHZ (B) after 7, 14, and 21 days of salt stress. The different letters are significant differences according to Duncan’s new multiple range test (*P* < 0.05) based on one-way ANOVA.

Compared with the control, the soluble protein content of X900 increased by 5.34%, 2.07%, and 2.38% after 7, 14, and 21 days of salt stress, respectively, and the soluble protein content of HHZ leaves increased by 1.31%, 2.83%, and 6.16%, respectively. Notably, under NaCl stress, foliar application of Pro-Ca increased the soluble protein content of X900 leaves by 0.4%, 5.72%, and 7.65% and HHZ by 1.79%, 1.34%, and 11.23%, respectively (Figs. 8A and 8B).

## Discussion

### Morphological parameters

Tillers are an important agronomic characteristic of rice growth and development (Zhao et al., 2020a; Zhao et al., 2020b; Ishikawa et al., 2005; Zhang et al., 2018). In intensive planting, a smaller tiller angle will make a compact plant structure, which may increase plant density and improve photosynthetic efficiency (Taier et al., 2021). A wider tiller angle can reduce humidity to avoid diseases, but an excessively large tiller angle will cover more space and may lead to a decrease in photosynthesis (Wang & Li, 2008; Rashid et al., 2022). Gao et al. (2018) speculated that the wider tillering angle of ZmPIF3 transgenic plants was due to more tillering growth. The results of this study show that exogenous foliar application of Pro-Ca can increase the tillering angles of two rice varieties under NaCl stress. This may be due to the asymmetric distribution of GA caused by exogenous foliar application of Pro-Ca, which leads to the differential expression of GA-regulated genes in the leaf sheath base of rice, resulting in the loosening of the cell wall at the leaf sheath base, therefore affecting the growth angle of rice tillers (Cui et al., 2005) (Table 1). In addition, we found that the tillering angle of the two rice varieties decreased under 0.3% NaCl stress compared with the control, but whether salt stress affects the tillering angle and the specific mechanism need to be further verified. The formation and elongation of rice tillering buds is also an important link in the tillering process (Shao et al., 2019; Wang & Li, 2011). In this study, we found that foliar application of Pro-Ca can effectively alleviate the inhibition of NaCl on rice tillering bud formation (Figs. 2A and 2B). Previous studies have proven that GA and cytokinin (CTK) play an antagonistic role in tillering, and the inhibitory effect of exogenous GA on tillering is the same as that accumulated in plants (Zhuang et al., 2019). Therefore, we speculate that Pro-Ca may promote the growth of rice tillering buds by inhibiting the synthesis of GA in tillering nodes and increasing the content of CTK.

It is worth noting that 0.3% NaCl stress (approximately 50 mM) is equivalent to low-concentration NaCl stress in pot experiments. Previous studies have drawn different conclusions about the effect of this concentration of NaCl stress on rice growth. It has been proven that 50 mM NaCl stress reduces plant height, tillering, leaf relative RWC, chlorophyll and yield (Naim, 2014). Khanam et al. (2018) and others also proved that 50 mM salt stress in pot experiments will have negative effects on the plant height, tiller number, leaf number and leaf area of rice. However, in the experiment of Hariadi et al. (2015), we found that 50 mM salt stress could not significantly inhibit the growth of some salt-tolerant varieties. In this experiment, we found that under 0.3% NaCl stress, the tiller number per plant, plant height, stem base width, leaf area, shoot dry weight (SDW), and root dry weight (RDW) of the two rice varieties were significantly lower than those under CK, and the inhibitory effect on HHZ was stronger than that on X900. This result is similar to that of Hakim et al. (2014) on rice and Zhang et al. (2021) on wheat. Previous studies have shown that NaCl stress can reduce the rice tillering number and plant height of rice at the tillering stage (Zeng & Shannon, 2000; Wang, Fang & Wang, 2015). Therefore, we speculate that even at the same salt concentration, different growth environments, different treatment periods and cultivation methods may have different effects on plants. Additionally, the application of Pro-Ca reduced the damage of 0.3% NaCl stress to rice root length, stem base width, leaf area, SDW, and RDW and effectively reduced the plant height and enhanced the lodging resistance of rice plants. This is similar to the conclusion reached by Kim et al. (2007) on rice. This result indicated that Pro-Ca might play a positive role in improving the salt tolerance of rice at the tillering stage.

### Photosynthetic capacity

Photosynthesis can transform light energy into organic matter, and its physiological process is sensitive to external changes (Chaves, Flexas & Pinheiro, 2009). Maintaining photosynthesis is an important mechanism for plants to adapt to salt tolerance (Fan et al., 2007). Long-term NaCl stress can lead to ionic toxicity and premature senescence of leaves, therefore reducing photosynthesis and nutrient accumulation (Munns & Tester, 2008). In this experiment, the *Pn*, *Gs*, *Tr*, and *Ci* of the two rice varieties decreased significantly under 0.3% NaCl stress. This is because salt stress leads to physiological drought of plants, and plants try to reduce their own *Gs* to minimize water loss, resulting in a decrease in the net assimilation amount of CO_2_, which cannot meet the demand of normal photosynthesis (Suzuki et al., 2012). Among them, the decrease in *Ci* and the decrease in *Pn* and *Gs* at the same time indicate that salt stress leads to a decrease in *Pn* through stomatal limitation in this experiment (Yang et al., 2006; Tang et al., 2018). Compared with the S treatment, the *Pn*, *Gs*, *Tr*, and *Ci* of the two rice varieties treated with Pro-Ca+S increased significantly (Fig. 5). The increase in *Tr* enhanced the water absorption and transportation capacity of plants, which was beneficial to improve photosynthesis and salt tolerance (Fan et al., 2007). Feng et al. (2021) reported that exogenous foliar application of Pro-Ca on soybean significantly increased chlorophyll content, increased *Pn*, and maintained photosynthetic processes under saline-alkali stress. Exogenous application of Pro-Ca can enhance the photosynthetic activity and adaptability to salt stress of rice mesophyll cells at the tillering stage. Similar to the results of this experiment, the results show that Pro-Ca can maintain the photosynthetic activity of rice leaves at the tillering stage under NaCl stress and enhance the stress resistance of rice at the tillering stage.

It is worth noting that in the previous discussion, we mentioned that rice has a smaller tillering angle under 0.3% NaCl stress, but its photosynthetic capacity is also significantly lower than that of the control. Li et al. (2021) suggested that too large or too small of a tillering angle will have adverse effects on rice yield, and proper tillering angle is very important for rice growth and yield. In this study, we theorized that too small a tillering angle may also affect the photosynthetic capacity of rice. Additionally, the main reason for the decrease in photosynthetic capacity is the physiological damage to rice caused by NaCl stress.

### Antioxidant enzymes and the indices of membrane damage

SOD, POD, APX and CAT, as essential antioxidant enzymes in the plant antioxidant enzyme system, can effectively remove reactive oxygen species produced by NaCl stress (Apel & Hirt, 2004). However, under different concentrations of salt stress, antioxidant enzymes will show different changes in activity. Mishra, Bhoomika & Dubey (2013) proved that the activities of SOD, Cu/Zn-SOD, and APX in salt-tolerant rice varieties increased with increasing salt concentration. In salt-sensitive seedlings, the activities of guaiacol peroxidase (GPX), CAT, monodehydroascorbate reductase (MDHAR), dehydroascorbate reductase (DHAR) and glutathione reductase (GR) increased under moderate salinity but decreased at higher salinity. In this study, compared with the control, the activities of SOD, CAT, APX, and POD increased under 0.3% NaCl stress. Exogenous foliar application of Pro-Ca can significantly increase the activities of SOD, POD, CAT, and APX in the two rice varieties at the tillering stage under NaCl stress, which indicates that Pro-Ca can enhance the activities of related antioxidant enzymes in rice leaves, reduce oxidative damage and improve the salt tolerance of rice at the tillering stage. Feng et al. (2021) also found that the application of exogenous Pro-Ca significantly increased the activities of antioxidant enzymes (SOD, CAT, and POD) in soybean seedlings under saline-alkali stress. An early study also showed that Pro-Ca can increase the activities of SOD, CAT, and POD enzymes by stimulating the antioxidant defense system of cucumber plants (Başak, 2021). However, with the extension of treatment time, the ROS scavenging ability of Pro-Ca in rice leaves at the tillering stage was weakened under NaCl stress, which may be due to its short-term effect (Kim et al., 2007). Environmental stress leads to oxidative damage to the cell membrane (Suzuki et al., 2012). As an index of membrane lipid peroxidation, MDA content and electrolyte leakage reflect the permeability change and damage degree of crop leaf peroxidation (Farooq et al., 2019). The exogenous foliar application of Pro-Ca made the MDA content and electrolyte leakage of the two rice varieties significantly lower than that of the S treatment (Fig. 6), which indicated that exogenous foliar application of Pro-Ca reduced the cell membrane damage caused by NaCl stress.

**Figure 9:**
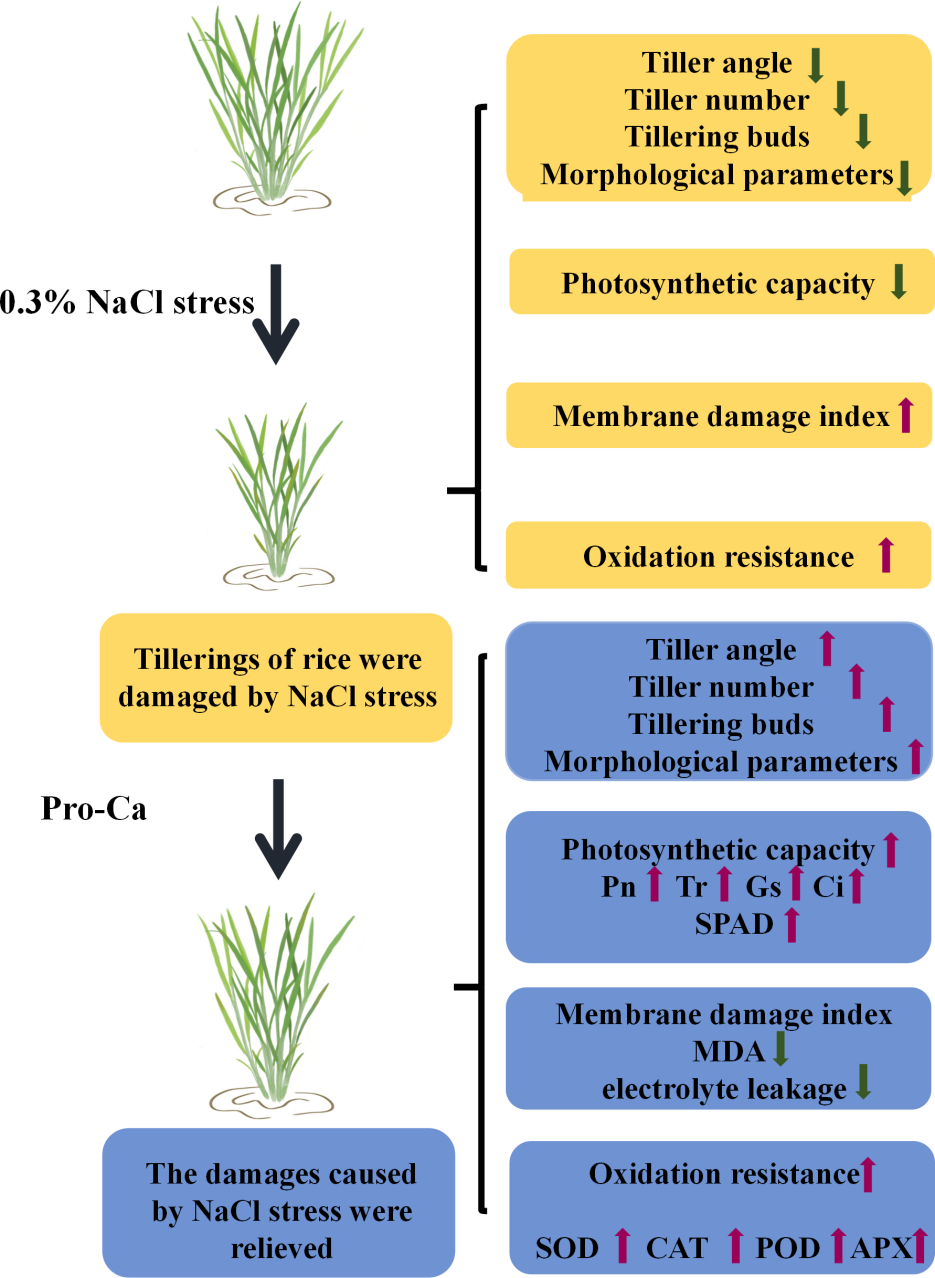
A proposed model shows the damage of NaCl stress to rice tillering and the improving effect of Pro-Ca, with the upward arrows indicate promotive effects and the downward arrows inhibitive effects.

### Osmotic substances

The accumulation of osmotic substances plays an important role in the activities of osmotic adjustment, carbon storage, free radical scavenging and cell membrane stabilization in plants, and osmotic substances can improve tolerance to various abiotic stresses by maintaining the reducing environment in plants (Dawood, Zaid & Latef, 2022). In this experiment, under 0.3% NaCl stress, the soluble protein content in the leaves of X900 and HHZ increased at the tillering stage (Fig. 8), which indicated that salt stress activated the osmotic adjustment substances of the plants, which had a regulatory and protective effect on the plants (Liu et al., 2018). This is also consistent with the research results of Ali et al. (2021). Exogenous foliar application of Pro-Ca under NaCl stress significantly increased the soluble protein content in the leaves of the two rice varieties, which indicated that Pro-Ca improved the osmotic adjustment ability of rice leaves, therefore improving the tolerance to salt stress. Previous studies have shown that Na^+^, K^+^, Ca^2+^, and Cl^−^ also participate in the regulation and mitigation of osmotic stress (Yang, Ding & Du, 2009). However, whether ions participate in osmotic regulation of rice leaves under salt stress in this experiment needs further study.

In summary, this article aims to clarify the resistance of the exogenous Pro-Ca to physiological and metabolic damage caused by NaCl stress before NaCl stress and provide a theoretical basis for enhancing rice lodging resistance and stress-resistant cultivation (Fig. 9).

## Conclusion

In this study, the protective effects of Pro-Ca on rice growth at the tillering stage under NaCl stress were examined. The results showed that 0.3% NaCl stress at the tillering stage could affect the tillering angle, growth of tillering buds, photosynthesis, and membrane lipid peroxidation of rice. Additionally, exogenous foliar application of Pro-Ca improves salt tolerance of rice, and reduces the damage of NaCl stress on rice morphology and physiological process at the tillering stage. However, the mechanism underlying Pro-Ca’s role in regulating hormone levels and ion distributions at the tillering stage under NaCl stress needs further study.

##  Supplemental Information

10.7717/peerj.14804/supp-1 Data S1 Raw data: the morphological and physiological characteristics of two rice varieties were damaged by NaCl stress and the alleviating effect of Pro-Ca. The mean ± standard error (SE), and different lowercase letters in the same column indicate that the mean values of replicates were significantly different among the treatments (*p* < 0.05). HHZ: Huanghuazhan rice cultivars. X900: Xiangliangyou900 rice cultivars.

## Additional Information and Declarations

### Data Availability

The following information was supplied regarding data availability: The raw measurements are available in the Supplementary Files.
